# Mortality outcomes in diabetic metabolic dysfunction-associated fatty liver disease: non-obese versus obese individuals

**DOI:** 10.1038/s41598-024-61896-5

**Published:** 2024-05-17

**Authors:** Pengwei Zhang, Yijun Zeng, Sijia Yang, Chunhong Ye, Mingwei Wang, Tianfang Peng, Li Li, Xianhui Dong

**Affiliations:** 1 Affiliated Hospital of Hangzhou Normal University, Hangzhou Normal University, Hangzhou, 310015 China; 2 Hangzhou Normal University, Hangzhou, 310015 China

**Keywords:** Metabolic (dysfunction)-associated fatty liver disease, Obesity, Normal weight, Type 2 diabetes mellitus, Mortality, Non-alcoholic fatty liver disease, Diabetes, Obesity

## Abstract

The difference in the survival of obese patients and normal-weight/lean patients with diabetic MAFLD remains unclear. Therefore, we aimed to describe the long-term survival of individuals with diabetic MAFLD and overweight/obesity (OT2M), diabetic MAFLD with lean/normal weight (LT2M), MAFLD with overweight/obesity and without T2DM (OM), and MAFLD with lean/normal weight and without T2DM (LM). Using the NHANESIII database, participants with MAFLD were divided into four groups. Hazard ratios (HRs) and 95% confidence intervals (CIs) for all-cause, cardiovascular disease (CVD)-related, and cancer-related mortalities for different MAFLD subtypes were evaluated using Cox proportional hazards models. Of the 3539 participants, 1618 participants (42.61%) died during a mean follow-up period of 274.41 ± 2.35 months. LT2M and OT2M had higher risks of all-cause mortality (adjusted HR, 2.14; 95% CI 1.82–2.51; *p* < 0.0001; adjusted HR, 2.24; 95% CI 1.32–3.81; *p* = 0.003) and CVD-related mortality (adjusted HR, 3.25; 95% CI 1.72–6.14; *p* < 0.0001; adjusted HR, 3.36; 95% CI 2.52–4.47; *p* < 0.0001) than did OM. All-cause and CVD mortality rates in LT2M and OT2M patients were higher than those in OM patients. Patients with concurrent T2DM and MAFLD should be screened, regardless of the presence of obesity.

## Introduction

Metabolic dysfunction-associated fatty liver disease (MAFLD) is the most common chronic liver disease worldwide^1^. Its prevalence in the United States increased from 22% in 1988–1994 to 36% in 2017–2020^2^. Moreover, MAFLD has more pathological phenotypes than its old definition, non-alcoholic fatty liver disease (NAFLD). In addition, it is associated with higher incidences of comorbid diseases such as diabetes, hypertension, and advanced fibrosis^3^ and can lead to increased all-cause mortality^4^, substantially compromising human health.

All-cause and cardiovascular disease (CVD)-related mortalities vary between different subtypes of MAFLD, and studies have shown higher risks of all-cause and disease-specific mortalities in patients with concurrent diabetes and MAFLD^5^. However, it is worth noting that several studies have reported the characteristics of all-cause and cause-specific mortalities of different subtypes categorized by normal body mass, overweight/obesity, and presence of type 2 diabetes mellitus (T2DM)^4–6^. In these studies, diabetic MAFLD included individuals with obesity and those with lean/normal weight. Previous studies have provided evidence for a possible obesity paradox regarding T2DM^7^; however, to the best of our knowledge, the difference in the survival of obese and normal-weight/lean patients with diabetic MAFLD remains unclear.

Therefore, this study aimed to investigate the mortality risk of different MAFLD subtypes—categorized based on body mass index (BMI) and the presence of T2DM—and provide a reference for personalized interventions for different MAFLD subtypes to improve the survival rate among diverse populations affected with MAFLD.

## Patients and methods

### Study population and design

**Figure 1:**
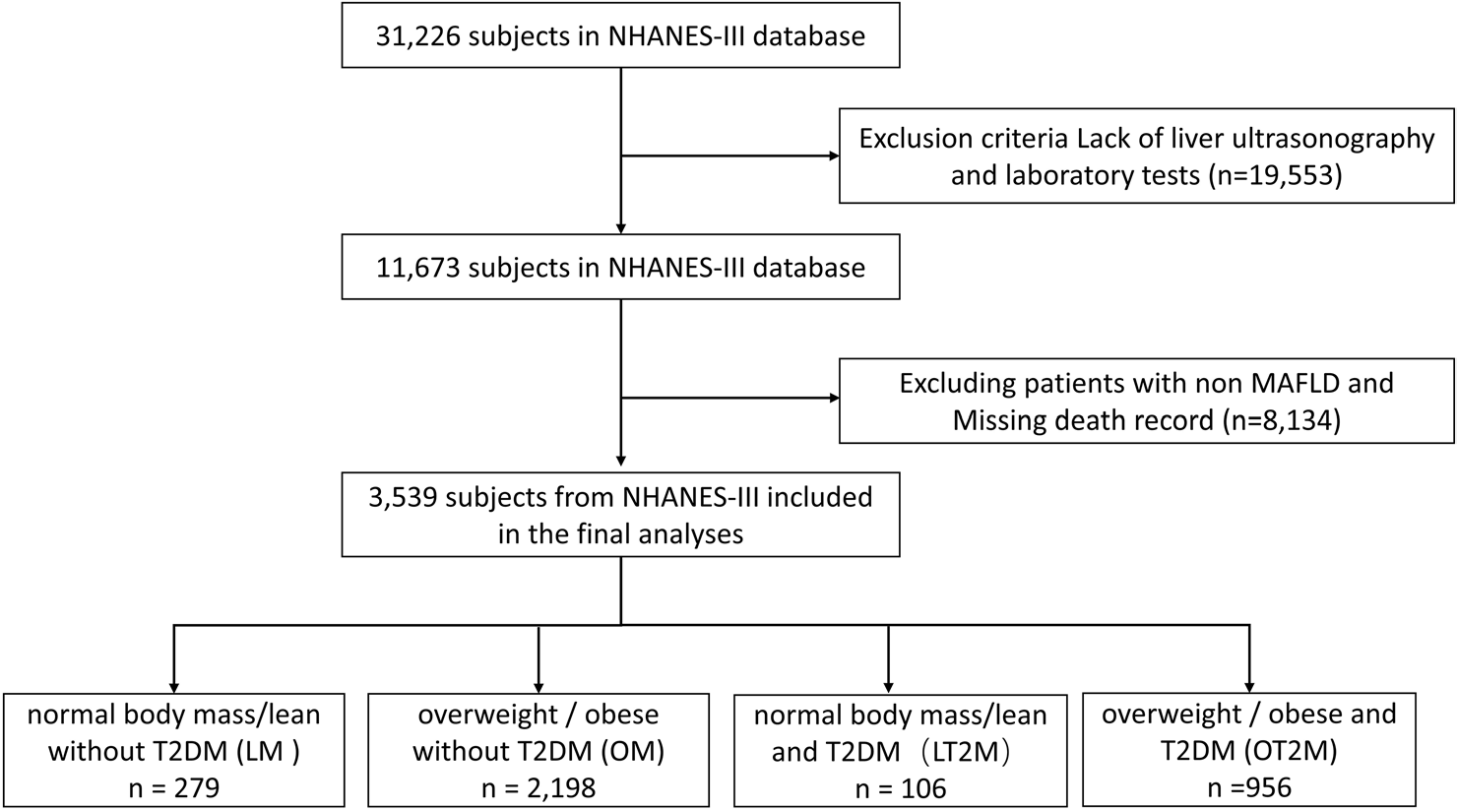
Flowchart of patients’ selection.

Raw data for this study were obtained from the Third National Health and Nutrition Examination Survey (NHANES III; 1988–1994) database, which includes liver ultrasound results; the collected data included patients’ demographics, questionnaire, examination, and laboratory data. The NHANES is a public database that is regularly surveyed on a nationally representative sample by the National Center for Health Statistics of the National Center for Disease Control and Prevention. Simultaneously, from the Center for Disease Control and Prevention, we obtained data on death as of December 31, 2019, that corresponded to the NHANES III data; these data included follow-up time, survival status, and leading underlying causes of death. Among the causes of death, we focused on CVDs and malignant tumors. Cause-specific mortality was defined using the International Classification of Diseases, Tenth Revision (ICD-10), codes: I00–I09, I11, I13, I20–I51, and I60–I69 were used for cardiovascular disease; C00–C97 for cancer. We selected 11,673 participants who had undergone liver ultrasound examinations among 31,226 participants. After excluding participants with incomplete data, we included data from 3539 participants with MAFLD (Fig. 1).

### Ethics approval and consent to participate

The Institutional Review Board of the Centers for Disease Control and Prevention approved the NHANSES III investigation scheme (https://www.cdc.gov/nchs/nhanes/irba98.htm), with written consent from the participants.

### Diagnostic criteria and definitions

In the NHANES III database, MAFLD is diagnosed based on the evidence of liver steatosis assessed by ultrasound combined with the presence of one of the following three criteria: overweight/obesity, T2DM, or metabolic dysfunction^8^. Overweight/obesity was defined as a BMI ≥ 25 kg/m^2^ in the examination data. T2DM was defined based on diabetes noted in the household adult data files, receiving medications for diabetes, or glycosylated hemoglobin A1c (HbA1c) ≥ 6.5% as observed from laboratory data. Metabolic dysfunction was primarily defined as a BMI < 25 kg/m^2^ and the presence of at least two risk factors for metabolic abnormalities, which were extracted from examination, household adult, and laboratory data. These risk factors included (1) waist circumference ≥ 102 cm or ≥ 88 cm for men and women, respectively; (2) blood pressure ≥ 130/85 mmHg or treatment with specific medications; (3) plasma triglyceride levels ≥ 150 mg/dL (≥ 1.70 mmol/L) or treatment with specific medications; (4) plasma high-density lipoprotein (HDL) cholesterol levels < 40 mg/dL (< 1.0 mmol/L) or < 50 mg/dL (< 1.3 mmol/L) for men or women, respectively, or treatment with specific medications; (5) presence of precursors of diabetes (HbA1c 5.7%–6.4% [39–47 mmol/mol]); and (6) plasma C-reactive protein (CRP) levels > 2 mg/L^8^. Fasting blood glucose levels and homeostasis model assessment of insulin resistance (HOMA-IR) were not available for data collection.

Hypertension was defined as the presence of hypertension as noted in household adult data files, receiving medications for hypertension, or examination data such as systolic blood pressure (SBP) ≥ 130 mmHg and diastolic blood pressure (DBP) ≥ 85 mmHg.

Regarding the degree of liver fibrosis in patients with MAFLD, the fibrosis-4 (FIB-4) score and NAFLD fibrosis score (NFS) were calculated^9,10^.

### Groups and variables

As shown in Fig. 1, patients with MAFLD were classified into four subtypes, based on their BMI and the presence of T2DM, as follows: (1) participants with LM were defined as those who had MAFLD with lean/normal weight (BMI < 25 kg/m^2^) and without T2DM; (2) participants with OM were defined as those who had MAFLD with overweight/obesity (BMI ≥ 25 kg/m^2^) and without T2DM; (3) participants with LT2M were defined as those who had MAFLD with lean/normal weight (BMI < 25 k g/m^2^) and T2DM; (4) participants with OT2M were defined as those who had MAFLD with overweight/obesity (BMI ≥ 25 kg/m^2^) and T2DM. Data regarding age, sex, race, smoking and drinking statuses, history of diabetes and hypertension, and medication history were collected from the NHANES III household adult data files. The following data were collected from the examination data: BMI, waist circumference, waist-to-hip ratio (WHR), SBP, and DBP. Blood measurement data were extracted from laboratory data and included high-sensitivity CRP, HbA1c, blood glucose (BG), insulin (INS), aspartate aminotransferase (AST), alanine transaminase (ALT), total cholesterol, triglyceride (TG), albumin, total bilirubin (T-Bil), high-density lipoprotein cholesterol, blood urea nitrogen (BUN), serum creatinine (SCr), C-Peptide (C-P), uric acid (UA), alkaline phosphatase, lactate dehydrogenase (LDH), and total serum protein.

### Statistical analyses

The NHANES III uses a complex, multi-stage probability sampling design to select participants representing the civil, non-institutionalized US population; therefore, sampling weights were used in all statistical analyses. Continuous data were expressed as means and standard errors, and categorical variables were expressed as percentages. One-way analysis of variance and Kruskal–Wallis tests were used to compare the data between groups. Subsequently, for variables with significant differences in the previous analyses, the LM group was used as a reference and the differences in data between the other three groups and the LM group were compared using a regression analysis. The Kaplan–Meier method was used to estimate the cumulative risk (cumulative incidence) of mortality for each MAFLD subtype. We used the Cox regression model to estimate the hazard ratios (HRs) and 95% confidence intervals (CIs) for the mortality rate of different subtypes, with LM as the reference. We used three models: model 1 was adjusted for age, sex, and race; model 2 was adjusted for age, sex, race, drinking, and smoking; and model 3 was adjusted for AST/ALT, TG, BUN, LDH, SCr, CRP, and hypertension. Subsequently, Cox regression analysis was conducted using OM, LT2M, and OT2M as references, respectively. Data were extracted and analyzed using R 4.1.2.0 (Vienna, Austria). The function cox.zph was used to test the proportional hazards assumption for a Cox regression model fit. We used the false-discovery rate (FDR) correction to adjust for multiple tests in the regression models. All Cox regression models met the proportional hazards assumption. *p*-values for all statistical analyses were bilateral, and the statistical significance was set at *p* < 0.05.

## Results

### Baseline characteristics of participants

This analysis included 3539 participants with an average age of 47.77 (0.42) years, of whom 55.75% were men and 44.25% were women. The median follow-up time was 274.41 ± 2.35 months. Table 1 shows the baseline characteristics of the study participants categorized according to BMI and presence of T2DM. The prevalence of LM, OM, LT2M, and OT2M was 7.52%, 68.30%, 2.31%, and 21.87%, respectively. In the analysis of different subtypes of MAFLD, compared with the LM group, the OT2M group had older individuals (53.68 vs. 47.69, *p* < 0.001), higher incidence of hypertension (71.33% vs. 56.07%, *p* = 0.007), higher incidence of hyperlipidemia (83.02% vs. 73.57%, *p* = 0.033), and higher levels of TG (2.68 vs. 2.02, *p* < 0.001), CRP (0.68 vs. 0.40, *p* < 0.001), BUN (15.35 vs. 13.90, *p* = 0.012), and TBil (0.63 vs. 0.57, *p* = 0.025). Patients in the LT2M and OT2M groups had higher levels of HbA1c and BG compared with patients in the LM group, and patients in the OM, LT2M, and OT2M groups had a higher prevalence of metabolic syndrome, higher C-P, higher INS, and more severe NFS than patients in the LM group. Notably, the AST/ALT levels in the LM group were higher than those in the OM and OT2M groups, and although OT2M and OM groups had higher UA levels than the LM group (OT2M vs. LM, 362.16 vs. 332.60,* p* = 0.001; OM vs. LM, 356.40 vs. 332.60, *p* = 0.007), the LT2M group had lower UA levels than the LM group (300.28 vs. 332.60, *p* = 0.011).

**Table 1:** Comparison of baseline characteristics of participants with different subtypes of MAFLD.

Variable	Total	LM	OM	LT2M	OT2M	*p* value	*p*		
							OM vs. LM	LT2M vs. LM	OT2M vs. LM
N, weighted prevalence	3539	279 (7.52%)	2198 (68.30%)	106 (2.31%)	956 (21.87%)				
Age, years	47.77 (0.42)	47.69 (1.21)	45.89 (0.45)	47.65 (2.38)	53.68 (0.76)	< 0.0001	0.166	0.988	< 0.001
Sex, %						0.46			
Female	1756 (44.25)	139 (48.07)	1075 (43.00)	50 (44.53)	492 (46.78)				
Male	1783 (55.75)	140 (51.93)	1123 (57.00)	56 (55.47)	464 (53.22)				
Race, %						< 0.0001	< 0.001	< 0.001	< 0.001
Black	828 (9.39)	66 (9.83)	496 (8.44)	30 (14.13)	236 (11.70)				
White	2565 (85.97)	195 (76.35)	1618 (88.05)	69 (67.45)	683 (84.73)				
Other	146 (4.64)	18 (13.81)	84 (3.51)	7 (18.42)	37 (3.57)				
HDL-C, mmol/L	1.15 (0.01)	1.21 (0.03)	1.15 (0.02)	1.24 (0.05)	1.11 (0.02)	0.01	0.079	0.624	0.006
BMI, kg/m^2^	30.85 (0.22)	22.84 (0.20)	31.25 (0.22)	23.06 (0.25)	33.19 (0.30)	< 0.0001	< 0.001	0.371	< 0.001
AST/ALT	1.20 (0.03)	1.47 (0.05)	1.19 (0.03)	1.30 (0.07)	1.15 (0.03)	< 0.0001	< 0.001	0.058	< 0.001
FIB-4	1.04 (0.04)	1.02 (0.04)	0.97 (0.04)	0.95 (0.06)	1.26 (0.10)	0.06			
NFS	− 1.92 (0.04)	− 2.72 (0.12)	− 2.18 (0.04)	− 2.08 (0.19)	− 0.82 (0.10)	< 0.0001	0.001	0.012	< 0.001
TC, mmol/L	5.55 (0.04)	5.52 (0.09)	5.51 (0.04)	5.59 (0.20)	5.70 (0.07)	0.21			
TG, mmol/L	2.27 (0.05)	2.02 (0.12)	2.17 (0.05)	2.22 (0.23)	2.68 (0.15)	0.01	0.244	0.476	< 0.001
CRP, mg/dL	0.49 (0.02)	0.40 (0.05)	0.44 (0.02)	0.47 (0.06)	0.68 (0.05)	< 0.0001	0.497	0.302	< 0.001
UA, μmol/L	354.57 (2.31)	332.60 (7.94)	356.40 (2.21)	300.28 (11.05)	362.16 (5.42)	< 0.0001	0.007	0.011	0.001
BUN, mmol/L	14.51 (0.13)	13.90 (0.35)	14.31 (0.13)	14.48 (0.54)	15.35 (0.33)	0.02	0.266	0.400	0.012
TBiL, mg/dL	0.61 (0.01)	0.57 (0.02)	0.61 (0.02)	0.53 (0.03)	0.63 (0.02)	0.02	0.176	0.363	0.025
SCr, μmol/L	96.68 (0.51)	96.49 (1.63)	96.55 (0.60)	97.12 (2.01)	97.10 (1.33)	0.97			
AST, U/L	24.42 (0.47)	22.75 (0.88)	24.35 (0.61)	22.53 (2.41)	25.38 (0.84)	0.13			
ALT, U/L	23.84 (0.69)	19.06 (1.66)	23.97 (0.81)	20.78 (2.23)	25.43 (1.01)	0.01	0.009	0.573	0.003
LDH, U/L	166.75 (2.28)	160.64 (2.93)	167.96 (2.61)	154.87 (5.59)	166.32 (2.49)	0.003	0.013	0.377	0.090
ALP, U/L	88.15 (0.94)	86.60 (2.17)	86.17 (1.05)	98.16 (6.93)	93.80 (1.64)	< 0.001	0.860	0.080	0.004
TP, g/dL	7.36 (0.02)	7.45 (0.04)	7.33 (0.02)	7.72 (0.12)	7.36 (0.02)	< 0.0001	0.005	0.048	0.045
ALB, g/L	41.51 (0.22)	41.92 (0.41)	41.66 (0.24)	41.12 (0.73)	40.93 (0.22)	< 0.0001	0.500	0.296	0.010
HbA1c, %	5.68 (0.03)	5.34 (0.05)	5.27 (0.02)	6.93 (0.29)	6.93 (0.10)	< 0.0001	0.171	< 0.001	< 0.001
BG, mmol/L	6.01 (0.05)	5.24 (0.03)	5.24 (0.02)	8.56 (0.48)	8.41 (0.18)	< 0.0001	0.959	< 0.001	< 0.001
C-P, nmol/L	1.05 (0.02)	0.70 (0.03)	0.97 (0.02)	0.99 (0.09)	1.40 (0.03)	< 0.0001	< 0.001	0.002	< 0.001
INS, pmol/L	16.94 (0.59)	9.83 (0.40)	14.58 (0.43)	19.18 (2.81)	26.54 (1.57)	< 0.0001	< 0.001	0.002	< 0.001
DBP, mmHg	78.29 (0.26)	76.53 (0.88)	78.65 (0.31)	76.52 (1.29)	77.98 (0.58)	0.05	0.029	0.997	0.090
Waist, cm	103.71 (0.49)	85.39 (0.76)	104.21 (0.50)	87.26 (0.83)	110.16 (0.59)	< 0.0001	< 0.001	0.060	< 0.001
Hipline, cm	176.00 (67.01)	92.55 (0.48)	208.65 (98.49)	92.92 (0.59)	111.49 (0.68)	< 0.0001	0.245	0.623	< 0.001
WHR	0.96 (0.00)	0.92 (0.01)	0.96 (0.00)	0.94 (0.01)	0.99 (0.00)	< 0.0001	< 0.001	0.103	< 0.001
MetS score	2.62 (0.04)	1.77 (0.09)	2.37 (0.04)	2.50 (0.16)	3.70 (0.06)	< 0.0001			
Smoking, %						0.01	0.024	0.410	< 0.001
Smoker	801 (23.95)	99 (31.46)	500 (24.25)	32 (37.56)	170 (18.99)				
Non-smoker	2738 (76.05)	180 (68.54)	1698 (75.75)	74 (62.44)	786 (81.01)				
Drinking, %						0.55			
Drinker	205 (5.60)	22 (5.26)	125 (5.12)	5 (7.75)	53 (6.99)				
Non-drinker	3334 (94.40)	257 (94.74)	2073 (94.88)	101 (92.25)	903 (93.01)				
Hypertension, %						< 0.0001	0.810	0.973	0.007
Hypertension	2085 (58.56)	155 (56.07)	1175 (54.81)	62 (56.34)	693 (71.33)				
Normal	1454 (41.44)	124 (43.93)	1023 (45.19)	44 (43.66)	263 (28.67)				
Hyperlipidemia, %						0.01	0.776	0.332	0.033
Hyperlipidemia	2630 (76.20)	193 (73.57)	1588 (74.66)	65 (66.02)	784 (83.02)				
Normal	909 (23.80)	86 (26.43)	610 (25.34)	41 (33.98)	172 (16.98)				
MetS, %						< 0.0001	< 0.001	< 0.001	< 0.001
MetS	1909 (54.46)	44 (20.77)	999 (48.46)	52 (53.02)	814 (84.94)				
Normal	1630 (45.54)	235 (79.23)	1199 (51.54)	54 (46.98)	142 (15.06)				
Fib-4 category, %						< 0.0001	0.049	0.424	0.083
Fib-4 < 1.4	2724 (79.46)	204 (77.38)	1789 (82.80)	75 (81.84)	656 (69.48)				
Fib-4 ≥ 1.4	815 (20.54)	75 (22.62)	409 (17.20)	31 (18.16)	300 (30.52)				
NFS category, %						< 0.0001	0.047	0.028	< 0.001
NFS < − 1.455	2168 (64.71)	220 (78.84)	1564 (72.89)	58 (65.00)	326 (34.30)				
NFS ≥ − 1.455	1371 (35.29)	59 (21.16)	634 (27.11)	48 (35.00)	630 (65.70)				
Time, month	274.41 (2.35)	269.82 (8.40)	288.39 (2.16)	229.79(15.06)	232.96 (5.66)	< 0.0001	0.014	0.040	< 0.001
Status, %						< 0.0001	0.035	0.246	0.003
Alive	1921 (57.39)	149 (55.22)	1397 (64.60)	32 (44.80)	343 (36.95)				
Deceased	1618 (42.61)	130 (44.78)	801 (35.40)	74 (55.20)	613 (63.05)				

MAFLD, metabolic dysfunction-associated fatty liver disease; LM, MAFLD with normal body mass/lean and without type 2 diabetes mellitus; OM, MAFLD with overweight/obesity and without type 2 diabetes mellitus; LT2M, MAFLD with normal body mass/lean and type 2 diabetes mellitus; OT2M, MAFLD with overweight/obesity and type 2 diabetes mellitus; HDL-C, High-density lipoprotein cholesterol; BMI, body mass index; FIB-4, fibrosis 4 score; NFS, NAFLD fibrosis score; APRI, Aspartate aminotransferase-to-Platelet Ratio Index; TC, total cholesterol; TG, triglyceride; CRP, C-reactive protein; UA, Uric acid; BUN, Blood urea nitrogen; TBiL, total bilirubin; SCr, blood creatinine; AST, aspartate amino-transferase; ALT, alanine aminotransferase; LDH, lactate dehydrogenase; ALP, alkaline phosphatase; TP, Total protein; ALB, albumin; HbA1c, glycosylated hemoglobin; BG, blood glucose; C-P, C-Peptide; INS, insulin; DBP, diastolic blood pressure; WHR, waist-to-hip ratio; MetS, metabolic syndrome.

### All-cause mortality for different subtypes of MAFLD

**Figure 2:**
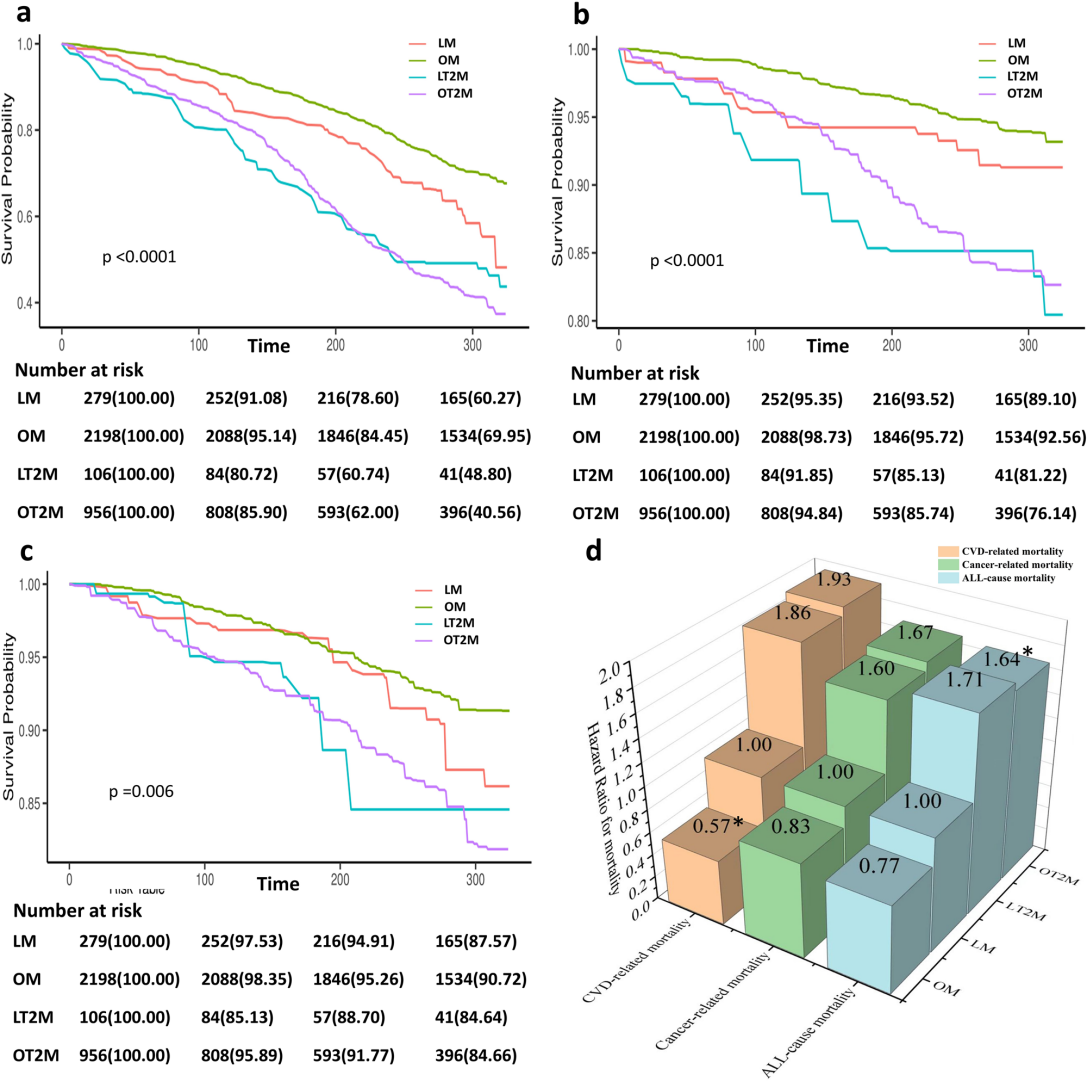
(**a**–**c**) Cumulative incidence of all-cause death, cardiovascular death, cancer death with different subtypes of MAFLD. Outcomes for (**a**) all-cause death, (**b**) cardiovascular death, and (**c**) cancer death. (**d**) Correlation between different subtypes of MAFLD and all-cause, cardiovascular-related, and cancer-related mortalities, using LM group as a reference (value of 1.00). Outcomes after adjusting for aspartate amino-transferase/alanine aminotransferase, triglyceride, blood urea nitrogen, lactate dehydrogenase, blood creatinine, C-reactive protein, and hypertension.

During a follow-up period of 274.41 (2.35) months, 1618 (42.61%) deaths occurred, and there were significant differences in mortality between the groups (log-rank test, *p* < 0.001; Fig. 2a). Using the LM group as a reference, all-cause mortality in the OM group was lower (unadjusted HR, 0.73; 95% CI 0.55–0.93;* p* = 0.01; Table 2) and the all-cause mortality in the OT2M group was 1.73 times higher than that in the LM group (unadjusted HR, 1.73; 95% CI 1.26–2.73;* p* < 0.001). These outcomes remained true even after adjusting for other variables (Table 2, Fig. 2d).

**Table 2:** Unadjusted and adjusted hazard ratios for all-cause, CVD-related, and cancer-related mortalities in patients with different subtypes of MAFLD.

Outcomes	Number of deaths/total, %	Unadjusted		Model 1		Model 2		Model 3	
		HR (95% CI)	*p*-value	HR (95% CI)	*p*-value	HR (95% CI)	*p*-value	HR (95% CI)	*p*-value
ALL-cause mortality									
LM	130/279 (44.78)	Ref		Ref		Ref		Ref	
OM	801/2198 (35.40)	0.73 (0.55–0.93)	0.01	0.73 (0.56–0.94)	0.01	0.77 (0.59–1.00)	0.052	0.77 (0.59–1.00)	0.046
LT2M	74/106 (55.20)	1.51 (0.90–2.53)	0.12	1.59 (0.98–2.57)	0.06	1.63 (1.03–2.58)	0.04	1.71 (1.04–2.83)	0.03
OT2M	613/956 (63.05)	1.73 (1.26–2.37)	< 0.001	1.38 (1.01–1.73)	0.04	1.44 (1.09–1.90)	0.01	1.64 (1.22–2.19)	< 0.001
CVD-related mortality									
LM	32/279 (12.49)	Ref		Ref		Ref		Ref	
OM	209/2198 (7.63)	0.55 (0.35–0.85)	0.01	0.43 (0.26–0.72)	0.001	0.48 (0.29–0.81)	0.01	0.57 (0.37–0.89)	0.01
LT2M	24/106 (16.56)	1.61 (0.76–3.41)	0.21	1.24 (0.56–2.75)	0.59	1.41 (0.65–3.05)	0.38	1.86 (0.89–3.91)	0.1
OT2M	180/956 (20.83)	2.18 (1.27–3.74)	0.005	1.21 (0.74–1.98)	0.45	1.44 (0.84–2.45)	0.18	1.93 (1.16–3.21)	0.01
Cancer-related mortality									
LM	32/279 (10.40)	Ref		Ref		Ref		Ref	
OM	198/2198 (9.33)	0.78 (0.47–1.30)	0.34	0.70 (0.37–1.30)	0.25	0.72 (0.40–1.30)	0.28	0.83 (0.49–1.40)	0.48
LT2M	12/106 (11.95)	1.43 (0.53–3.86)	0.48	1.43 (0.48–4.27)	0.52	1.43 (0.50–4.10)	0.5	1.60 (0.56–4.54)	0.38
OT2M	116/956 (11.26)	1.59 (0.86–2.92)	0.14	1.11 (0.59–2.06)	0.75	1.18 (0.64–2.16)	0.59	1.67 (0.93–3.01)	0.09

Model 1 was adjusted for age, sex and race; model 2 was adjusted for age, sex, race, drinking, and smoking; and model 3 was adjusted for AST/ALT, TG, BUN, LDH, SCr, CRP, and hypertension.

Data are expressed as hazard ratios (HRs) and 95% confidence intervals (95% CIs).

MAFLD, metabolic dysfunction-associated fatty liver disease; LM, MAFLD with normal body mass/lean and without type 2 diabetes mellitus; OM, MAFLD with overweight/obesity and without type 2 diabetes mellitus; LT2M, MAFLD with normal body mass/lean and type 2 diabetes mellitus; OT2M, MAFLD with overweight/obesity and type 2 diabetes mellitus; CVD, cardiovascular disease; AST, aspartate amino-transferase; ALT, alanine aminotransferase; TG, triglyceride; BUN, Blood urea nitrogen; LDH, lactate dehydrogenase; SCr, blood creatinine; CRP, C-reactive protein.

Subsequently, we used the OM, LT2M, and OT2M groups as references (Table 3), and the results showed that the all-cause mortality in the LM group was higher than that in the OM group (adjusted HR, 1.31; 95% CI 1.00–1.70; *p* = 0.046), whereas the all-cause mortality in the OT2M group was higher than that in the OM group (adjusted HR, 2.14; 95% CI 1.82–2.51; *p* < 0.0001). The all-cause mortality in the LT2M group was 2.42 times higher than that in the OM group (adjusted HR, 2.24; 95% CI 1.32–3.81; *p* = 0.003).

**Table 3:** Paired comparison of all-cause, CVD-related, and cancer-related mortalities between the different MAFLD subtypes.

Outcomes	Unadjusted		Model 1		Model 2		Model 3	
	HR (95% CI)	*p*-value	HR (95% CI)	*p*-value	HR (95% CI)	*p*-value	HR (95% CI)	*p*-value
ALL-cause mortality								
LM vs. OM	1.38 (1.07–1.77)	0.015	1.38 (1.07–1.78)	0.02	1.30 (1.00–1.69)	0.06	1.31 (1.00–1.70)	0.06
LT2M vs. OM	2.08 (1.21–3.58)	0.015	2.19 (1.32–3.63)	0.006	2.11 (1.30–3.43)	0.009	2.24 (1.32–3.81)	0.006
OT2M vs. OM	2.38 (2.02–2.81)	0.0012	1.83 (1.58–2.12)	0.0006	1.87 (1.62–2.14)	0.0006	2.14 (1.82–2.51)	0.006
OT2M vs. LT2M	1.15 (0.69–1.91)	0.6	0.83 (0.53–1.31)	0.43	0.88 (0.57–1.36)	0.57	0.95 (0.60–1.53)	0.85
CVD-related mortality								
LM vs. OM	1.83 (1.17–2.86)	0.015	2.33 (1.40–3.89)	0.015	2.07 (1.24–3.47)	0.015	1.74 (1.12–2.71)	0.015
LT2M vs. OM	2.94 (1.44–6.03)	0.006	2.90 (1.45–5.82)	0.006	2.93 (1.51–5.68)	0.002	3.25 (1.72–6.14)	0.002
OT2M vs. OM	4.00 (2.96–5.40)	0.0006	2.82 (2.25–3.55)	0.0006	2.98 (2.37–3.75)	0.0006	3.36 (2.52–4.47)	0.0006
OT2M vs. LT2M	1.36 (0.64–2.88)	0.43	0.97 (0.49–1.92)	0.94	1.02 (0.54–1.93)	0.96	1.30 (0.53–2.01)	0.92
Cancer-related mortality								
LM vs. OM	1.28 (0.77–2.12)	0.51	1.43 (0.77–2.67)	0.375	1.38 (0.77–2.48)	0.42	1.21 (0.72–2.04)	0.576
LT2M vs. OM	1.83 (0.70–4.77)	0.44	2.05 (0.78–5.39)	0.30	1.98 (0.78–5.04)	0.3	1.93 (0.71–5.25)	0.4
OT2M vs. OM	2.03 (1.40–2.95)	0.006	1.59 (1.14–2.22)	0.06	1.63 (1.19–2.22)	0.012	2.02 (1.40–2.94)	0.006
OT2M vs. LT2M	1.11 (0.42–2.94)	0.83	0.77 (0.30–1.98)	0.59	0.82 (0.32–2.10)	0.68	1.05 (0.40–2.72)	0.92

Model 1 was adjusted for age, sex and race; model 2 was adjusted for age, sex, race, drinking, and smoking; and model 3 was adjusted for AST/ALT, TG, BUN, LDH, SCr, CRP, and hypertension.

Data are expressed as hazard ratios (OR) and 95% confidence intervals (95% CI).

MAFLD, metabolic dysfunction-associated fatty liver disease; LM, MAFLD with normal body mass/lean and without type 2 diabetes mellitus; OM, MAFLD with overweight/obesity and without type 2 diabetes mellitus; LT2M, MAFLD with normal body mass/lean and type 2 diabetes mellitus; OT2M, MAFLD with overweight/obesity and type 2 diabetes mellitus; CVD, cardiovascular disease; AST, aspartate amino-transferase; ALT, alanine aminotransferase; TG, triglyceride; BUN, Blood urea nitrogen; LDH, lactate dehydrogenase; SCr, blood creatinine; CRP, C-reactive protein.

All *p* were FDR-adjusted.

### Cancer- and CVD-related mortalities for different subtypes of MAFLD

During a follow-up period of 274.41 (2.35) months, 445 CVD-related (12.57%) and 358 cancer-related deaths (10.12%) occurred. There were significant differences in CVD-related mortality between the different subtypes (log-rank test, *p* < 0.001; Fig. 2b), and the OM group had the lowest risk of CVD-related mortality. When the LM group was used as a reference, the OM group had lower CVD-related mortality (adjusted HR, 0.57; 95% CI 0.37–0.89; *p* = 0.01; Fig. 2d). CVD-related mortality in the OT2M group was 1.93 times higher than that in the LM group (adjusted HR, 1.93; 95% CI 1.16–3.21; *p* = 0.01; Fig. 2d). A subsequent pairwise comparative analysis showed that LT2M and OT2M were associated with a higher risk of CVD-related mortality compared with OM alone (adjusted HR, 3.25; 95% CI 1.72–6.14; *p* < 0.0001; adjusted HR, 3.36; 95% CI 2.52–4.47; *p* < 0.0001; Table 3). There were no significant differences in Cancer-related mortality between the different subtypes (log-rank test, *p* = 0.16; Fig. 2c), but we found that the risk of cancer-related death in the OT2M group was 2.02 times higher than that in the OM group (adjusted HR, 2.02; 95% CI 1.40–2.94; *p* < 0.0001; Table 3).

MAFLD: metabolic dysfunction-associated fatty liver disease, LM: MAFLD with normal body mass/lean and without type 2 diabetes mellitus, OM: MAFLD with overweight/obesity and without type 2 diabetes mellitus, LT2M: MAFLD with normal body mass/lean and type 2 diabetes mellitus, OT2M: MAFLD with overweight/obesity and type 2 diabetes mellitus.

## Discussion

This cohort study, with a follow-up period of more than 20 years, aimed to elucidate the relationship between all-cause, CVD-related, and cancer-related mortalities in different subtypes of MAFLD, which were categorized in terms of BMI and presence of T2DM. The results showed that LM, LT2M, and OT2M had higher risks of all-cause and CVD-related mortalities than did OM. OT2M had a higher all-cause mortality than did LM, and OT2M was associated with a higher risk of cancer-related mortality than OM.

MAFLD is a metabolic disease that often coexists with diabetes, and both are mutually causal^11^. T2DM also accelerates the progression of liver disease in NAFLD, affects the survival of patients with NAFLD^12^, and increases all-cause and CVD-related mortalities in these patients^11^; this situation did not change with the revision of the definition from NAFLD to MAFLD. A recent study suggested that patients with concurrent diabetes and MAFLD might have a higher risk of all-cause mortality^6^. This is also corroborated by our study, which showed that compared with normal-weight patients with MAFLD, individuals with concurrent obesity, diabetes, and MAFLD or normal-weight patients with concurrent diabetes and MAFLD had higher all-cause and CVD-related mortalities, with CVD-related death being the leading cause of death among patients with MAFLD^13^. In a previous study, with neither FLD (patients with overlapping NAFLD and MAFLD) as a reference, patients with concurrent diabetes mellitus and MAFLD had higher odds of coronary artery calcification (CAC) than did patients with MAFLD in other groups (odds ratio, 5.833; 95% CI 3.047–11.164 for CAC scores > 100). Additionally, patients with concurrent diabetes mellitus and MAFLD had higher insulin levels^14^. Insulin resistance, a common pathophysiological mechanism in diabetes mellitus, obesity, and MAFLD, increases vascular stiffness and consequently promotes CVD progression^5,15^. Insulin resistance can also alter whole-body lipid metabolism, which may in turn lead to the development of dyslipidemia and the lipid triad. Together with endothelial dysfunction, which can also be induced by aberrant insulin signaling, insulin resistance may contribute to atherosclerotic plaque formation^16^.

Our study focused on lean/normal-weight patients with diabetic MAFLD because up to 20% of individuals with diabetes have a normal weight^17^, and MAFLD needs to be examined in lean patients with T2DM^18^. When we analyzed the data of patients with MAFLD combined with T2DM according to BMI, we found that the LT2M group had higher risks of CVD-related mortality than the OM group. The subsequent pairwise comparison of the LT2M and OT2M groups did not show any significant differences in all-cause or CVD-related mortalities, suggesting that patients with normal body mass/lean diabetic MAFLD have a higher risk of death, similar to patients with obese diabetic MAFLD.

MAFLD is closely associated with obesity^19^, and studies have shown that obesity increases all-cause and CVD-related mortalities in patients with MAFLD^20^. However, there is increasing evidence that patients with overweight/obesity and MAFLD have lower mortality rates than normal-weight patients^21^. This phenomenon has been termed the “obesity paradox,” which has been extensively discussed in the context of CVDs and chronic kidney disease, and there is increasing discussion and evidence of it in the context of chronic liver diseases^22^. In the present study, we found higher all-cause and CVD-related mortalities in normal-weight patients with MAFLD than in those with obesity and MAFLD when T2DM was excluded as a factor. Several recent studies have reported similar results^6,23^. A U-shaped relationship between BMI and mortality was found in a cohort study in China, suggesting that lean patients with MAFLD might experience higher all-cause mortality^24^, and the influence of diabetes was not ruled out. The relationship between obesity and mortality is complex, and the mechanisms underlying the “obesity paradox” are difficult to elucidate^22,25^. A possible explanation for this is that steatotic livers in lean participants may be more vulnerable to liver injury despite a lower degree of hepatic steatosis, as suggested by studies using liver tissue biopsies that showed a higher proportion of patients with lobular inflammation in the lean NAFLD group than in the group comprising patients with overweight/obesity and NAFLD (*p* < 0.001)^26^. Furthermore, BMI has certain limitations when assessing obesity, with studies showing that hepatic and cardiovascular damage in lean patients with NAFLD is associated with visceral adiposity rather than BMI^27^. Visceral adiposity is a risk factor for CVDs and is associated with cancer^28^. An increase in visceral fat induces chronic local inflammation and simultaneously leads to adipose dysfunction; chronic inflammation is strongly associated with CVDs and cancer^29^. In addition, the definition of MAFLD requires metabolic abnormalities in a population with lean/normal weight; previous studies have shown an association between metabolically unhealthy status and a higher risk of MAFLD and the development of steatohepatitis and liver fibrosis, independent of BMI^1^. At the genetic level, genes such as PNPLA3, which is one of the first genes shown to be associated with NAFLD, seem to be important in NAFLD development in individuals without obesity^30^.

Our study had several limitations. First, the NHANES III database is primarily based on a US population-based sampling; thus, our findings may not be generalizable to other ethnic groups. In future studies, diverse ethnic populations should be included. Second, although we found differences in all-cause, CVD-related, and cancer-related mortalities between the different subtypes of MAFLD, the present cross-sectional study could not establish a causal relationship between all-cause, CVD-related, and cancer-related mortalities and the different subtypes of MAFLD. Third, because of the slow progression of MAFLD, only data on the initial body weights and prevalence of T2DM were obtained, and whether long-term changes in BMI had an impact on the outcome of MAFLD could not be assessed. Finally, the diagnosis of MAFLD was based on ultrasonography without using histology of liver biopsy specimens to determine the degree of hepatic steatosis and that of liver fibrosis. Because histological examination of liver biopsy specimens is performed for the diagnosis of MAFLD only in difficult or complicated cases^18^, it is not suitable for studies with large samples.

In conclusion, our study showed that all-cause and CVD-related mortalities varied between the different subtypes of MAFLD. Compared with the OM group, the LT2M and OT2M groups had higher all-cause and CVD-related mortalities, followed by the LM group. This remained true even after adjusting for other variables. With no currently available approved drugs, reducing obesity remains the primary recommendation for MAFLD. However, whether weight loss is also required in lean patients with MAFLD and patients with diabetic MAFLD should be considered. Given the heterogeneity of MAFLD, further studies are required to reclassify MAFLD, which is of great significance for precise therapeutic interventions.

## Data Availability

All data generated/analyzed during this study are included in this published article. The raw data can be found at https://www.cdc.gov/nchs/nhanes/index.htm and https://www.cdc.gov/nchs/data-linkage/mortality-public.htm.

## Abbreviations

ALT: Alanine transaminase
AST: Aspartate aminotransferase
BG: Blood glucose
BMI: Body mass index
BUN: Blood urea nitrogen
CAC: Coronary artery calcification
CI: Confidence interval
C-P: C-peptide
CVD: Cardiovascular disease
DBP: Diastolic blood pressure
FIB-4: Fibrosis-4
HDL: High-density lipoprotein
HOMA-IR: Homeostasis model assessment of insulin resistance
HbA1c: Glycosylated hemoglobin A1c
HR: Hazard ratio
INS: Insulin
LDH: Lactate dehydrogenase
MAFLD: Metabolic dysfunction-associated fatty liver disease
NAFLD: Non-alcoholic fatty liver disease
NFS: NAFLD fibrosis score
SBP: Systolic blood pressure
SCr: Serum creatinine
T2DM: Type 2 diabetes mellitus
T-Bil: Total bilirubin
TG: Triglyceride
UA: Uric acid
WHR: Waist-to-hip ratio
